# 2-Amino-5-methyl­pyridinium 3-chloro­benzoate

**DOI:** 10.1107/S1600536812043231

**Published:** 2012-10-24

**Authors:** Kaliyaperumal Thanigaimani, Abbas Farhadikoutenaei, Nuridayanti Che Khalib, Suhana Arshad, Ibrahim Abdul Razak

**Affiliations:** a School of Physics, Universiti Sains Malaysia, 11800 USM, Penang, Malaysia

## Abstract

The 3-chloro­benzoate anion of the title salt, C_6_H_9_N_2_^+^·C_7_H_4_ClO_2_^−^, is nearly planar with a dihedral angle of 2.44 (13)° between the benzene ring and the carboxyl­ate group. In the crystal, the protonated N atom and the 2-amino group of the cation are hydrogen bonded to the carboxyl­ate O atoms of the anion *via* a pair of N—H⋯O hydrogen bonds with an *R*_2_^2^(8) ring motif, forming an approximately planar ion pair with a dihedral angle of 7.92 (5)° between the pyridinium and benzene rings. The ion pairs are further connected *via* N—H⋯O and C—H⋯O hydrogen bonds, forming a two-dimensional network parallel to the *bc* plane.

## Related literature

For background to the chemistry of substituted pyridines, see: Pozharski *et al.* (1997 ▶); Katritzky *et al.* (1996 ▶). For details of hydrogen bonding, see: Jeffrey (1997 ▶); Scheiner (1997 ▶). For hydrogen-bond motifs, see: Bernstein *et al.* (1995 ▶). For bond-length data, see: Allen *et al.* (1987 ▶). For stability of the temperature controller used for the data collection, see: Cosier & Glazer (1986 ▶).
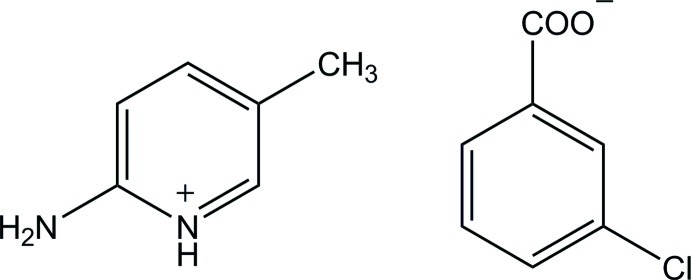


## Experimental

### 

#### Crystal data

C_6_H_9_N_2_^+^·C_7_H_4_ClO_2_^−^
*M* *_r_* = 264.70
Monoclinic, 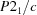

*a* = 9.0318 (11) Å
*b* = 11.6590 (14) Å
*c* = 12.1166 (15) Å
β = 101.521 (2)°
*V* = 1250.2 (3) Å^3^
*Z* = 4
Mo *K*α radiation
μ = 0.30 mm^−1^
*T* = 100 K
0.53 × 0.31 × 0.22 mm

#### Data collection

Bruker SMART APEXII DUO CCD area-detector diffractometer
Absorption correction: multi-scan (*SADABS*; Bruker, 2009 ▶) *T*_min_ = 0.856, *T*_max_ = 0.936
13594 measured reflections
3629 independent reflections
3201 reflections with *I* > 2σ(*I*)
*R*_int_ = 0.031

#### Refinement

*R*[*F* ^2^ > 2σ(*F* ^2^)] = 0.038
*wR*(*F* ^2^) = 0.115
*S* = 1.06
3629 reflections
176 parameters
H atoms treated by a mixture of independent and constrained refinement
Δρ_max_ = 0.48 e Å^−3^
Δρ_min_ = −0.33 e Å^−3^

### 

Data collection: *APEX2* (Bruker, 2009 ▶); cell refinement: *SAINT* (Bruker, 2009 ▶); data reduction: *SAINT*; program(s) used to solve structure: *SHELXTL* (Sheldrick, 2008 ▶); program(s) used to refine structure: *SHELXTL*; molecular graphics: *SHELXTL*; software used to prepare material for publication: *SHELXTL* and *PLATON* (Spek, 2009 ▶).

## Supplementary Material

Crystal structure: contains datablock(s) global, I. DOI: 10.1107/S1600536812043231/is5201sup1.cif

Structure factors: contains datablock(s) I. DOI: 10.1107/S1600536812043231/is5201Isup2.hkl

Supplementary material file. DOI: 10.1107/S1600536812043231/is5201Isup3.cml

Additional supplementary materials:  crystallographic information; 3D view; checkCIF report

## Figures and Tables

**Table 1:** Hydrogen-bond geometry (Å, °)

*D*—H⋯*A*	*D*—H	H⋯*A*	*D*⋯*A*	*D*—H⋯*A*
N1—H1*N*1⋯O1	1.00 (2)	1.68 (2)	2.6716 (13)	174 (2)
N2—H1*N*2⋯O2	0.946 (19)	1.820 (19)	2.7618 (15)	173.0 (19)
N2—H2*N*2⋯O1^i^	0.90 (2)	1.95 (2)	2.8526 (14)	174.0 (17)
C2—H2*A*⋯O2^ii^	0.95	2.52	3.2104 (15)	130

Symmetry codes: (i) 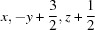; (ii) 
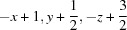
.

**Fig. 1.**
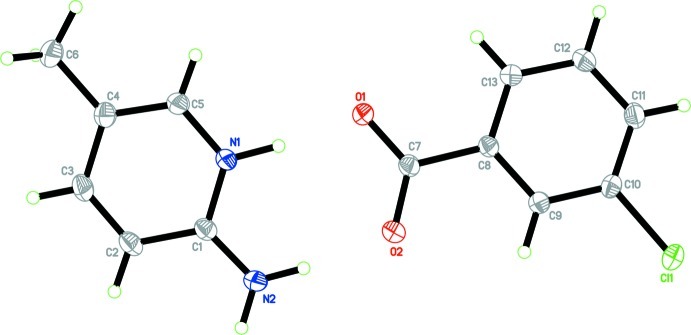
The molecular structure of the title compound with atom labels with 50% probability displacement ellipsoids.

**Fig. 2.**
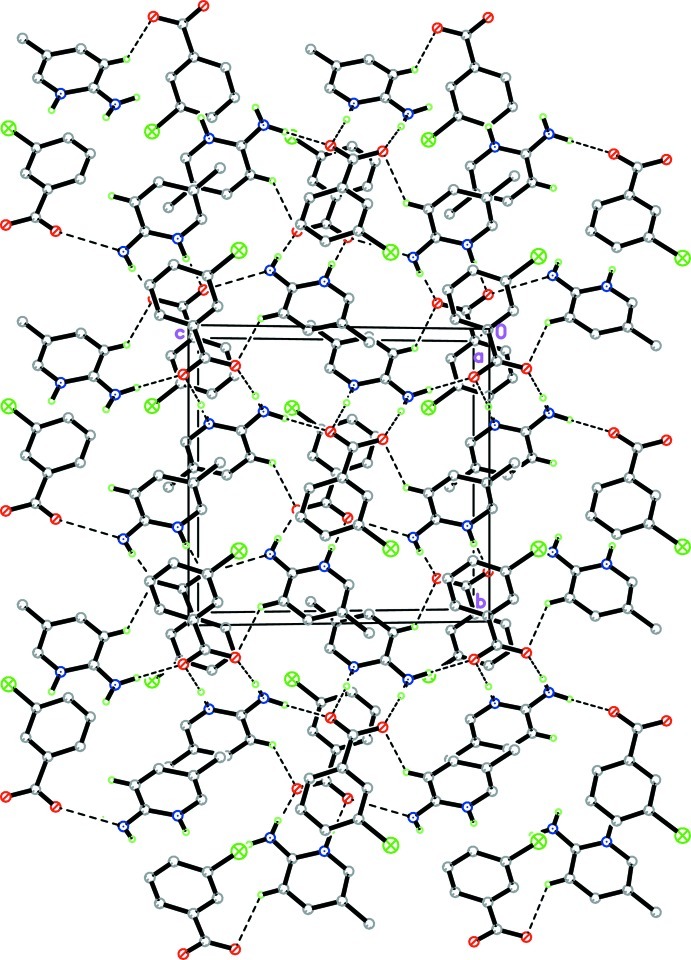
The crystal packing of the title compound, showing hydrogen-bonded (dashed lines) two-dimensional networks parallel to the bc plane.

## supplementary crystallographic information

### Comment

Pyridine and its derivatives play an important role in heterocyclic chemistry (Pozharski *et al.*, 1997; Katritzky *et al.*, 1996). They are often involved in hydrogen-bond interactions (Jeffrey, 1997; Scheiner, 1997). In order to study some interesting hydrogen bonding interactions, the synthesis and structure of the title compound, (I), is presented here.

The asymmetric unit of the title compound contains a protonated 2-amino-5-methylpyridinium cation and a 3-chlorobenzoate anion (Fig. 1). In the 2-amino-5-methylpyridinium cation, a wider than normal angle [C1—N1—C5 = 122.50 (10)°] is subtended at the protonated N1 atom. The 2-amino-5-methylpyridinium cation is planar with a maximum deviation of 0.001 (1) Å for atom C2. The dihedral angle between the pyridine (N1/C1–C5) and benzene (C8–C13) rings is 7.92 (5)°. The bond lengths (Allen *et al.*, 1987) and angles are normal. In the crystal packing (Fig. 2), the protonated N1 atom and a nitrogen atom of the 2-amino group (N2) are hydrogen-bonded to the carboxylate oxygen atoms (O1 and O2) *via* a pair of N—H···O hydrogen bonds (Table 1), forming a ring motif *R*_2_^2^(8) (Bernstein *et al.*, 1995). Furthermore, these motifs are connected *via* N2—H2N2···O1^i^ and C2—H2A···O2^ii^ hydrogen bonds to form a two-dimensional network parallel to the *bc* plane.

### Experimental

Hot methanol solutions (20 ml) of 2-amino5-methylpyridine (54 mg, Aldrich) and 3-chlorobenzoic acid (39 mg, Merck) were mixed and warmed over a heating magnetic stirrer hotplate for a few minutes. The resulting solution was allowed to cool slowly at room temperature and crystals of the title compound (I) appeared after a few days.

### Refinement

N-bound H atoms were located in a difference Fourier maps and refined freely [refined N—H distances 1.00 (2), 0.949 (19) and 0.90 (2) Å]. The remaining H atoms were positioned geometrically (C—H= 0.95 and 0.98 Å) and were refined using a riding model, with *U*_iso_(H) = 1.2 *U*_eq_(C) or 1.5*U*_eq_(methyl C). A rotating-group model was used for the methyl group. In the final refinement, four outliers were omitted (1 1 8, 1 0 8, 2 3 1 and -1 4 8).

### Crystal data

**Table:**

C_6_H_9_N_2_^+^·C_7_H_4_ClO_2_^−^	*F*(000) = 552
*M**_r_* = 264.70	*D*_x_ = 1.406 Mg m^−^^3^
Monoclinic, *P*2_1_/*c*	Mo *K*α radiation, λ = 0.71073 Å
Hall symbol: -P 2ybc	Cell parameters from 6768 reflections
*a* = 9.0318 (11) Å	θ = 2.3–30.1°
*b* = 11.6590 (14) Å	µ = 0.30 mm^−^^1^
*c* = 12.1166 (15) Å	*T* = 100 K
β = 101.521 (2)°	Block, colourless
*V* = 1250.2 (3) Å^3^	0.53 × 0.31 × 0.22 mm
*Z* = 4	

### Data collection

**Table:**

Bruker SMART APEXII DUO CCD area-detector diffractometer	3629 independent reflections
Radiation source: fine-focus sealed tube	3201 reflections with *I* > 2σ(*I*)
Graphite monochromator	*R*_int_ = 0.031
φ and ω scans	θ_max_ = 30.1°, θ_min_ = 2.3°
Absorption correction: multi-scan (*SADABS*; Bruker, 2009)	*h* = −12→12
*T*_min_ = 0.856, *T*_max_ = 0.936	*k* = −16→16
13594 measured reflections	*l* = −17→17

### Refinement

**Table:**

Refinement on *F*^2^	Primary atom site location: structure-invariant direct methods
Least-squares matrix: full	Secondary atom site location: difference Fourier map
*R*[*F*^2^ > 2σ(*F*^2^)] = 0.038	Hydrogen site location: inferred from neighbouring sites
*wR*(*F*^2^) = 0.115	H atoms treated by a mixture of independent and constrained refinement
*S* = 1.06	*w* = 1/[σ^2^(*F*_o_^2^) + (0.070*P*)^2^ + 0.2814*P*] where *P* = (*F*_o_^2^ + 2*F*_c_^2^)/3
3629 reflections	(Δ/σ)_max_ < 0.001
176 parameters	Δρ_max_ = 0.48 e Å^−^^3^
0 restraints	Δρ_min_ = −0.33 e Å^−^^3^

### Special details

**Table:**

Experimental. The crystal was placed in the cold stream of an Oxford Cryosystems Cobra open-flow nitrogen cryostat (Cosier & Glazer, 1986) operating at 100.0 (1) K.
Geometry. All e.s.d.'s (except the e.s.d. in the dihedral angle between two l.s. planes) are estimated using the full covariance matrix. The cell e.s.d.'s are taken into account individually in the estimation of e.s.d.'s in distances, angles and torsion angles; correlations between e.s.d.'s in cell parameters are only used when they are defined by crystal symmetry. An approximate (isotropic) treatment of cell e.s.d.'s is used for estimating e.s.d.'s involving l.s. planes.
Refinement. Refinement of *F*^2^ against ALL reflections. The weighted *R*-factor *wR* and goodness of fit *S* are based on *F*^2^, conventional *R*-factors *R* are based on *F*, with *F* set to zero for negative *F*^2^. The threshold expression of *F*^2^ > σ(*F*^2^) is used only for calculating *R*-factors(gt) *etc*. and is not relevant to the choice of reflections for refinement. *R*-factors based on *F*^2^ are statistically about twice as large as those based on *F*, and *R*- factors based on ALL data will be even larger.

### Fractional atomic coordinates and isotropic or equivalent isotropic displacement parameters (Å^2^)

**Table:**

	*x*	*y*	*z*	*U*_iso_*/*U*_eq_	
Cl1	1.05016 (3)	0.25195 (2)	0.66489 (3)	0.02535 (11)	
O1	0.60056 (10)	0.65207 (8)	0.46906 (7)	0.02385 (19)	
O2	0.72588 (10)	0.62484 (8)	0.64581 (7)	0.02350 (19)	
N1	0.45310 (10)	0.81988 (8)	0.55172 (8)	0.01776 (19)	
N2	0.55220 (12)	0.77783 (10)	0.73908 (9)	0.0219 (2)	
C1	0.46396 (12)	0.84318 (9)	0.66248 (10)	0.0179 (2)	
C2	0.37858 (13)	0.93693 (10)	0.69173 (10)	0.0210 (2)	
H2A	0.3833	0.9561	0.7686	0.025*	
C3	0.28968 (13)	0.99913 (10)	0.60827 (11)	0.0218 (2)	
H3A	0.2327	1.0615	0.6283	0.026*	
C4	0.27996 (12)	0.97334 (10)	0.49275 (10)	0.0204 (2)	
C5	0.36421 (12)	0.88246 (10)	0.46891 (10)	0.0190 (2)	
H5A	0.3607	0.8624	0.3924	0.023*	
C6	0.18156 (14)	1.04262 (11)	0.40224 (11)	0.0262 (3)	
H6A	0.1968	1.0170	0.3283	0.039*	
H6B	0.2084	1.1239	0.4124	0.039*	
H6C	0.0754	1.0321	0.4070	0.039*	
C7	0.69320 (12)	0.59788 (9)	0.54401 (9)	0.0175 (2)	
C8	0.76799 (11)	0.49160 (9)	0.50805 (9)	0.0159 (2)	
C9	0.86515 (12)	0.42923 (9)	0.59063 (9)	0.0172 (2)	
H9A	0.8840	0.4537	0.6670	0.021*	
C10	0.93380 (12)	0.33117 (9)	0.55982 (10)	0.0180 (2)	
C11	0.91118 (13)	0.29472 (10)	0.44867 (10)	0.0212 (2)	
H11A	0.9603	0.2280	0.4288	0.025*	
C12	0.81496 (13)	0.35797 (10)	0.36693 (10)	0.0224 (2)	
H12A	0.7987	0.3345	0.2904	0.027*	
C13	0.74215 (12)	0.45553 (10)	0.39638 (9)	0.0198 (2)	
H13A	0.6750	0.4973	0.3402	0.024*	
H1N1	0.511 (3)	0.7554 (18)	0.526 (2)	0.055 (7)*	
H1N2	0.617 (2)	0.7251 (16)	0.7129 (17)	0.037 (5)*	
H2N2	0.574 (2)	0.8015 (16)	0.8114 (16)	0.035 (4)*	

### Atomic displacement parameters (Å^2^)

**Table:**

	*U*^11^	*U*^22^	*U*^33^	*U*^12^	*U*^13^	*U*^23^
Cl1	0.02441 (17)	0.02607 (17)	0.02424 (17)	0.00991 (9)	0.00168 (12)	0.00262 (10)
O1	0.0302 (4)	0.0259 (4)	0.0161 (4)	0.0125 (3)	0.0061 (3)	0.0030 (3)
O2	0.0285 (4)	0.0253 (4)	0.0163 (4)	0.0075 (3)	0.0034 (3)	−0.0021 (3)
N1	0.0183 (4)	0.0195 (4)	0.0156 (4)	0.0034 (3)	0.0037 (3)	−0.0019 (3)
N2	0.0240 (5)	0.0262 (5)	0.0150 (5)	0.0050 (4)	0.0025 (4)	−0.0025 (4)
C1	0.0168 (5)	0.0201 (5)	0.0173 (5)	−0.0003 (4)	0.0047 (4)	−0.0028 (4)
C2	0.0206 (5)	0.0231 (5)	0.0203 (5)	0.0005 (4)	0.0066 (4)	−0.0061 (4)
C3	0.0193 (5)	0.0207 (5)	0.0263 (6)	0.0030 (4)	0.0064 (4)	−0.0047 (4)
C4	0.0183 (5)	0.0202 (5)	0.0230 (5)	0.0024 (4)	0.0045 (4)	0.0000 (4)
C5	0.0189 (5)	0.0215 (5)	0.0166 (5)	0.0021 (4)	0.0038 (4)	−0.0006 (4)
C6	0.0253 (6)	0.0258 (6)	0.0269 (6)	0.0084 (4)	0.0038 (5)	0.0024 (5)
C7	0.0195 (5)	0.0181 (5)	0.0163 (5)	0.0023 (4)	0.0069 (4)	0.0013 (4)
C8	0.0156 (4)	0.0169 (4)	0.0159 (5)	0.0012 (3)	0.0049 (4)	0.0000 (4)
C9	0.0166 (4)	0.0187 (5)	0.0164 (5)	0.0011 (4)	0.0037 (4)	0.0000 (4)
C10	0.0146 (4)	0.0188 (5)	0.0205 (5)	0.0019 (3)	0.0030 (4)	0.0016 (4)
C11	0.0197 (5)	0.0203 (5)	0.0238 (6)	0.0029 (4)	0.0046 (4)	−0.0039 (4)
C12	0.0242 (5)	0.0246 (5)	0.0182 (5)	0.0032 (4)	0.0036 (4)	−0.0047 (4)
C13	0.0208 (5)	0.0223 (5)	0.0160 (5)	0.0033 (4)	0.0029 (4)	−0.0003 (4)

### Geometric parameters (Å, º)

**Table:**

Cl1—C10	1.7447 (11)	C5—H5A	0.9500
O1—C7	1.2729 (13)	C6—H6A	0.9800
O2—C7	1.2498 (14)	C6—H6B	0.9800
N1—C1	1.3535 (14)	C6—H6C	0.9800
N1—C5	1.3645 (14)	C7—C8	1.5163 (15)
N1—H1N1	1.00 (2)	C8—C13	1.3914 (15)
N2—C1	1.3348 (15)	C8—C9	1.3961 (15)
N2—H1N2	0.949 (19)	C9—C10	1.3870 (15)
N2—H2N2	0.90 (2)	C9—H9A	0.9500
C1—C2	1.4227 (15)	C10—C11	1.3878 (16)
C2—C3	1.3671 (17)	C11—C12	1.3914 (16)
C2—H2A	0.9500	C11—H11A	0.9500
C3—C4	1.4171 (17)	C12—C13	1.3955 (16)
C3—H3A	0.9500	C12—H12A	0.9500
C4—C5	1.3686 (15)	C13—H13A	0.9500
C4—C6	1.5019 (16)		
			
C1—N1—C5	122.50 (10)	C4—C6—H6C	109.5
C1—N1—H1N1	121.5 (14)	H6A—C6—H6C	109.5
C5—N1—H1N1	116.0 (14)	H6B—C6—H6C	109.5
C1—N2—H1N2	117.5 (12)	O2—C7—O1	124.84 (10)
C1—N2—H2N2	119.1 (12)	O2—C7—C8	117.27 (9)
H1N2—N2—H2N2	119.4 (17)	O1—C7—C8	117.88 (10)
N2—C1—N1	119.33 (10)	C13—C8—C9	119.93 (10)
N2—C1—C2	122.91 (11)	C13—C8—C7	121.91 (9)
N1—C1—C2	117.75 (10)	C9—C8—C7	118.16 (9)
C3—C2—C1	119.39 (11)	C10—C9—C8	119.15 (10)
C3—C2—H2A	120.3	C10—C9—H9A	120.4
C1—C2—H2A	120.3	C8—C9—H9A	120.4
C2—C3—C4	121.97 (10)	C9—C10—C11	121.77 (10)
C2—C3—H3A	119.0	C9—C10—Cl1	118.45 (9)
C4—C3—H3A	119.0	C11—C10—Cl1	119.77 (8)
C5—C4—C3	116.45 (10)	C10—C11—C12	118.59 (10)
C5—C4—C6	122.35 (11)	C10—C11—H11A	120.7
C3—C4—C6	121.20 (10)	C12—C11—H11A	120.7
N1—C5—C4	121.94 (10)	C11—C12—C13	120.61 (11)
N1—C5—H5A	119.0	C11—C12—H12A	119.7
C4—C5—H5A	119.0	C13—C12—H12A	119.7
C4—C6—H6A	109.5	C8—C13—C12	119.93 (10)
C4—C6—H6B	109.5	C8—C13—H13A	120.0
H6A—C6—H6B	109.5	C12—C13—H13A	120.0
			
C5—N1—C1—N2	179.47 (10)	O2—C7—C8—C9	1.54 (15)
C5—N1—C1—C2	−0.06 (16)	O1—C7—C8—C9	−177.53 (10)
N2—C1—C2—C3	−179.39 (11)	C13—C8—C9—C10	−0.51 (16)
N1—C1—C2—C3	0.12 (16)	C7—C8—C9—C10	179.45 (9)
C1—C2—C3—C4	−0.16 (18)	C8—C9—C10—C11	1.46 (16)
C2—C3—C4—C5	0.13 (17)	C8—C9—C10—Cl1	−178.02 (8)
C2—C3—C4—C6	179.96 (11)	C9—C10—C11—C12	−1.01 (17)
C1—N1—C5—C4	0.03 (17)	Cl1—C10—C11—C12	178.47 (9)
C3—C4—C5—N1	−0.06 (16)	C10—C11—C12—C13	−0.39 (18)
C6—C4—C5—N1	−179.89 (10)	C9—C8—C13—C12	−0.85 (17)
O2—C7—C8—C13	−178.50 (10)	C7—C8—C13—C12	179.19 (10)
O1—C7—C8—C13	2.43 (16)	C11—C12—C13—C8	1.32 (18)

### Hydrogen-bond geometry (Å, º)

**Table:**

*D*—H···*A*	*D*—H	H···*A*	*D*···*A*	*D*—H···*A*
N1—H1*N*1···O1	1.00 (2)	1.68 (2)	2.6716 (13)	174 (2)
N2—H1*N*2···O2	0.946 (19)	1.820 (19)	2.7618 (15)	173.0 (19)
N2—H2*N*2···O1^i^	0.90 (2)	1.95 (2)	2.8526 (14)	174.0 (17)
C2—H2*A*···O2^ii^	0.95	2.52	3.2104 (15)	130

Symmetry codes: (i) *x*, −*y*+3/2, *z*+1/2; (ii) −*x*+1, *y*+1/2, −*z*+3/2.
